# Rural reticence to inform physicians of cannabis use

**DOI:** 10.1111/jrh.12885

**Published:** 2024-09-25

**Authors:** Daniel J. Mallinson, Timothy J. Servinsky

**Affiliations:** ^1^ School of Public Affairs Penn State Harrisburg Middletown Pennsylvania USA; ^2^ Center for Survey Research Penn State Harrisburg Middletown Pennsylvania USA

**Keywords:** cannabis, medical marijuana, rural opinion, survey research

## Abstract

**Purpose:**

Over 75% of Americans have legal access to medical cannabis, though physical access is not uniform and can be difficult for rural residents. Additionally, substantial stigma remains in using medical cannabis, particularly within the health care system. This article argues that rural Americans may be particularly affected by such stigma and may thus be more likely to not report cannabis use to health care providers.

**Methods:**

Data were obtained from 1,045 adult Pennsylvanians using a self‐administered web panel omnibus survey. Rurality was determined by overlaying Zip Code Tabulation Areas with urban areas, as defined by the U.S. Census Bureau. Primary outcomes were prior use of cannabidiol (CBD) or marijuana and reporting of such use to medical professionals. Covariates utilized in logistic regressions included rurality, gender, age, race/ethnicity, political affiliation, political ideology, and veteran status.

**Findings:**

Living in an urban area was positively associated with disclosure of marijuana use to health care providers as compared to those in rural areas, although there were no differences found in CBD disclosure.

**Conclusions:**

Stigma surrounding marijuana usage may have a disproportionate impact on health outcomes for rural residents who use marijuana. Nonreporting prevents effective holistic medical care and can result in negative drug interactions and other side effects.

## INTRODUCTION

Cannabis policy in the United States is a complex thicket for many actors to navigate. Lingering federal prohibition has complicated the implementation of comprehensive medical cannabis programs in 38 states and adult‐use recreational programs in 26, plus the District of Columbia.^1^ The harbinger of broader legalization, medical cannabis is technically within reach of over 75% of Americans,^2^ but access and utilization are not uniform. Moreover, the process of medicalizing cannabis, both in procedure and cultural acceptance, is incomplete.^3^ Rural Americans, in particular, have faced challenges in both accessing cannabis and in facing the stigma that remains among the public and within the health care system.^4^, ^5^ The closer citizens reside to dispensaries, the more likely they are to be certified, even for low‐evidence conditions, which disproportionally reduces access for those living in rural areas.^6^ But even if a rural patient can access a dispensary, they may have trouble finding a physician to recommend cannabis and may also be less willing to discuss their desire to use medical cannabis with their primary care physician (PCP), due to lingering stigma.^7^

While much is known about drug use and perceptions in rural areas, little is known specifically about the views of Americans living in rural areas regarding medical cannabis and their potential reluctance to discuss cannabis use with a physician. This study seeks to help fill this gap through a representative sample survey of Pennsylvanians conducted in 2023. The survey asked respondents about their views on medical and adult‐use cannabis, as well as their willingness to use medical cannabis and, if they have used it, to discuss such use with a physician or other health care professional. We find that urban residents are more likely to report using cannabis to their doctors than rural residents, but there is no difference in the use of cannabidiol (CBD). This is likely due to the difference in stigma between the two.

This study has implications for public health services and policy focused on rural areas. There can be serious consequences for individuals when they do not consider how medical cannabis fits into their overall health care strategy.^8^ After briefly reviewing the literature on cannabis and rurality, presenting the survey design and analytical methodology, and reporting the results, this manuscript considers what these findings mean as states like Pennsylvania consider expanding into adult‐use recreational cannabis and as the federal government contemplates broader reform.

## LITERATURE REVIEW

### Rural differences in substance use and abuse

While much attention has been paid to the growing opioid epidemic in both urban and rural America, the challenges facing rural areas are mounting. Rural adolescents, for example, are at a substantially higher risk of prescription opioid misuse than those from large urban areas.^9^ Mortality risks from drugs, alcohol, and suicide, so‐called “deaths of despair,” are higher in rural areas.^10^ Alcohol‐related problems are particularly prevalent among farmers.^11^ A study in rural Nebraska examined “ready access” to marijuana, methamphetamine, heroin, and prescription pills.^12^ Cannabis was most readily available at 35% of the sample, then prescription pills at 17.8%, methamphetamine at 8.9%, and finally heroin at 4.5%. Notably, Nebraska did not, and still does not, have a state‐legal medical or adult‐use recreational cannabis program, yet cannabis was still the substance with the most ready access. While older studies showed no difference in cannabis use between rural and urban youth, more recent studies of cannabis use among rural adolescents found it to be higher than among urban, as are the rates of abuse for other substances.^13^, ^14^, ^15^ Further, polysubstance use becomes more prevalent in high school for rural students.^16^ There are even significantly different misperceptions about the safety of driving after cannabis use. While rural teens in Montana were more familiar with drunk driving policies, they perceived driving after cannabis use to be less dangerous than after drinking.^17^

Even in states that have legalized cannabis either medicinally or recreationally, the purported benefits are not as strong for rural residents as for urban ones. The economic benefits of cannabis legalization are stronger in urban areas than in rural.^18^ The implementation of marijuana legalization is more vexing for less‐resourced rural police than for urban. Rural police officers are more likely to express concerns about the negative externalities associated with legalization and have fewer resources to address things like driving while high.^19^ They point to the general decline of rural areas as a culprit in these difficulties. Some research finds opioid misuse falls with greater access to state‐legal cannabis, but other studies show that these effects are not consistent geographically.^20^, ^21^

### The rural doctor‐patient relationship

Rural patients already face challenges with respect to the availability of health care services, availability of doctors, and generally poorer health.^22^ Rural Americans are also more likely to avoid health care.^23^ But the rural doctor‐patient relationship, when it exists, can be strong. Rural physicians tend to have more personal interactions with patients outside of the office. Greater socioemotional communication between rural doctors and patients can help build trust, as can the length of the doctor‐patient relationship.^24^, ^25^ There is evidence that greater patient‐doctor trust increases patients’ willingness to discuss medical cannabis with their doctors.^26^ Patients are also more likely to access medical cannabis *if* that trusted physician recommends it.^26^

But with a highly stigmatized substance like cannabis, a close relationship that includes socializing outside of the clinical setting could also become a hindrance. If patients are more likely to interact with their doctors outside of the clinical setting, they may not want them to know that they either use or are considering using cannabis medicinally or recreationally. This means that such use could remain unreported to a patient's PCP, which can have significant health impacts if the patient experiences negative interactions between cannabis and other modalities of care.^8^, ^27^ Many studies have demonstrated patient reticence in reporting cannabis use or interest in medical cannabis to health care providers due to both anticipated and experienced stigma.^7^ We argue that such reticence may be stronger for rural Americans, who have more‐frequent out‐of‐clinic interactions with their physicians, than for those living in urban or suburban areas.^24^

Nonreporting can have serious consequences. Cannabis has known interactions with many different medications.^8^, ^28^ Given that many patients wish to replace existing medications with cannabis, for example, for pain management, such replacement needs to be managed.^29^, ^30^ Veterans have even turned to forums like Reddit to gather information about using cannabis for self‐treatment rather than consult physicians at the Veterans Health Administration for fear of stigma.^31^ We turn now to testing whether rural residents are less likely to report cannabis use to their health care providers than urban ones.

## METHODS

### Data collection

To test our expectation, we used data from 1,045 self‐administered web panel responses that were collected by the Spring 2023 Lion Poll, an omnibus survey of adult Pennsylvanians administered biannually by [*name redacted for blind review*]. As of Spring 2023, Pennsylvania had an operating medical cannabis program, but not adult‐use recreational. Notably, the surrounding states of New Jersey and New York had operational adult‐use recreational programs. Neighboring Maryland did not implement adult use until after the survey period. The Lion Poll surveys Pennsylvanians on a variety of topics to measure public attitudes and track public policy issues for researchers and government agencies. The data were collected through Qualtrics web survey software between March 6 and April 2, 2023. All surveys were conducted in English, and the study was approved as exempt research by Penn State Institutional Review Board.

Waves of survey invitations were sent to potential survey respondents, adult (18+) Pennsylvanians who had completed a double opt‐in recruitment process with one of CSR's panel partners. The panel partners recruited panelists using a blended recruitment strategy from a variety of sources to identify respondents who do not join traditional panels. The partners also utilized a multistep validation process to ensure high‐quality responses. Respondents received a small incentive of between $1 and $8 for participating in the survey. Although respondents were compensated by panel partners, the research team never had access to respondents’ names, contact information, or other identifying information, and the identifying key resided with the panel partner only. Furthermore, the panel partners never had access to any survey data. Therefore, the data were collected anonymously.

To ensure that the results of the Lion Poll were not biased toward any location, age, or gender, a quota‐based invitation system was used to guarantee that the final dataset would be representative of the Commonwealth of Pennsylvania's known population by region (based on county) and, separately, by age and gender combined categories. State population estimates from the U.S. Census Bureau (updated July 1, 2021) were used to establish quotas. To minimize potential biases, key demographic responses were monitored and compared to Census data to ensure that the final sample was reflective of characteristics that are known to potentially skew representativeness. Rigorous strategies were employed to ensure high‐quality data, such as the inclusion of robust screening questions, attention checks and straight‐lining checks, internal validity checks for survey responses, and a thorough review of open‐ended responses and other quality indicators.

Respondents were not selected from the general population at random. Rather, only adults who opted to participate in a paid web survey panel were included in the sampling frame. In addition, the results are representative only of those who chose to participate. As a result, sample frame and nonresponses biases might prevent a direct comparison to Pennsylvania's general population.^32^

### Survey design

Respondents were asked several questions to better understand Pennsylvanians’ cannabinoid usage and reporting to health care providers. Respondents were first asked, “Have you ever heard of CBD, also known as cannabidiol?” Respondents who answered “yes” were then asked, “Have you ever tried or taken a product containing CBD, also known as cannabidiol?” Those who responded “yes” to this question were then asked, “Have you ever told your primary care physician, pharmacist, nurse, or other healthcare professional that you have tried or taken a non‐prescription product containing CBD?” Response options for each question included: yes, no, don't know/not sure, prefer to not answer.

All respondents were then asked, “Have you ever tried or taken marijuana or hashish, whether medically or for recreational purposes?” Those who answered “yes” were then asked 2 questions: “Have you ever had a medical marijuana card?” and “Have you ever told your primary care physician, pharmacist, nurse, or other healthcare professional that you have tried or taken marijuana?”

### Rurality

To categorize rurality for respondents’ zip codes, geographic boundary files for the U.S. Census Bureau's Zip Code Tabulation Areas (ZCTAs) and Urban Areas were analyzed using the Bureau's updated 2020 Urban Area definitions.^i^ This analysis was conducted by overlaying ZCTAs with Urban Areas to calculate the percentage of each ZCTA encompassing an Urban Area. Factors such as land cover, characterized by a significant degree of impervious surfaces, along with housing and population density, and the contiguity of these urbanized areas, are considered by the U.S. Census Bureau in its classification of Urban Areas. The urban/rural percentage values for each ZCTA were then mapped to its constituent ZIP Codes. This mapping was facilitated through the latest (2019) ZIP Code to ZCTA Crosswalk file provided by UDS Mapper, a tool of the Health Resources & Services Administration (HRSA) under the Uniform Data System (UDS).^ii^ Consequently, a value reflecting the percentage of land classified as an Urban Area by the U.S. Census Bureau was assigned to each ZIP Code.

### Statistical analysis

An initial sample size of 1,045 was chosen to allow for sufficient power to identify differences between regions, with the smallest region having 72 respondents. The survey had a margin of sampling error of ± 3.0%. The survey's response rate of 2.5% was calculated using the American Association of Public Opinion Research's (AAPOR) Response Rate 3 (RR3) formula. RR3 is obtained by dividing the number of completed interviews by the sum of the numbers of completed interviews, partially completed interviews, refusals, and noncontacts. The participation rate is then adjusted by estimating the proportion of cases of unknown eligibility based on the known proportion of eligible cases of all cases for which eligibility was determined. The cooperation rate was 87.0% using AAPOR's Cooperation Rate 3 (CR3) formula. CR3 is calculated by dividing the number of completed interviews by the sum of the numbers of completed interviews, partially completed interviews, and refusals. Of the 1,238 respondents who answered all of the questions preceding the cannabis question series, 89 ended their participation while answering the cannabis questions, resulting in a break‐off rate of 7.2% during the cannabis question series. This may have contributed to the survey's lower response rate. Data were extracted from the Qualtrics Online Survey Platform into Statistical Package for the Social Sciences software (version 29.0; IBM SPSS Statistics) to process and document the dataset.

Differences were first assessed by comparing responses between rural and urban survey participants (at the zip code level) using chi‐square and *t*‐tests. Logistic regression analyses were then conducted to examine these relationships while controlling for several covariates, described in the next section. Results were reported at *P* <.05, assuming a 95% confidence level.

### Covariates

The covariates used in our analyses included age (continuous, and then categorical when indicated), gender (man, woman, other), rurality (by zip code), veteran status, race/ethnicity (Hispanic, non‐Hispanic White, non‐Hispanic Other), political affiliation (Democrat, Republican, other), and political ideology (liberal, conservative, other).

## RESULTS

All survey respondents provided zip codes, resulting in a final valid sample size of 1,045. Of these respondents, 49.5% lived in a rural zip code and 50.5% lived in an urban zip code. Rural respondents were more likely than urban respondents to be registered as Republicans (39.1% vs 23.1% for urban respondents) or not registered to vote (19.9% vs 10.8%), conservative (38.9% vs 24.6%), and non‐Hispanic White (91.9% vs 74.4%). There were no differences in gender, age, or veteran status by rurality (Table 1).

**TABLE 1:** Demographic characteristics by respondent zip code rurality.

Characteristics	Rural	Urban	*P* value
N	517 (49.5)	528 (50.5)	
Woman	274 (53.0)	261 (49.4)	.25014
18‐34	132 (25.5)	155 (29.4)	.16758
35‐64	253 (48.9)	255 (48.3)	.83366
65 and older	132 (25.5)	118 (22.3)	.22628
Veteran	46 (8.9)	33 (6.3)	.1031
Republican	**200 (39.1**)	**120 (23.1)**	**<.00001**
Democrat	**139 (27.1)**	**276 (53.1)**	**<.00001**
Other political party	71 (13.9)	68 (13.1)	.71138
Not registered to vote	**102 (19.9)**	**56 (10.8)**	**<.00001**
Conservative	**188 (38.9)**	**125 (24.6)**	**<.00001**
Moderate	182 (37.7)	204 (40.2)	.42372
Liberal	**113 (23.4)**	**179 (35.2)**	**<.00001**
White, non‐Hispanic	**475 (91.9)**	**392 (74.4)**	**<.00001**
Black or African American, non‐Hispanic	**4 (0.8)**	**74 (14)**	**<.00001**
Something else, non‐Hispanic	17 (3.3)	32 (6.1)	.03318
Hispanic	21 (4.1)	29 (5.5)	.27572

*Note*: Variables with missing data include: veteran status (n = 1), political party affiliation (n = 11), political ideology (n = 54), and race/ethnicity (n = 1).

Overall, 94.2% of respondents (n = 1,045) had heard of CBD. Of those who had heard of CBD (n = 984), 48.8% had tried or taken a product containing CBD at some point. Of those who had tried or taken CBD (n = 480), 52.1% have told a PCP, pharmacist, nurse, or other health care provider that they had tried or taken it. Those living in urban zip codes were more likely to have told a health care professional that they had tried or taken CBD, 56.6% versus 47.1%, *P* = .046.

Additionally, 59.7% of respondents (n = 1,045) had tried or taken marijuana, whether medically or recreationally, and 12.8% have had a medical marijuana card. There were no significant differences in medical marijuana reporting between those living in rural and urban zip codes, 11.3% versus 14.5%, *P* = .119. Of those who had tried or taken marijuana and chose to answer (n = 586), 53.9% have told a PCP, pharmacist, nurse, or other health care provider that they had tried or taken it. Those living in an urban zip code were more likely to say that they had tried or taken marijuana in the last year, 34.5% versus 25.6%, *P* = .002, and that they had told a health care professional about such use, 59.6% versus 47.7%, *P* = .004. Respondents were also more likely to report that they had tried or taken marijuana than CBD, 59.7% versus 48.8%, *P* < .001, and that they had tried or taken marijuana longer than a year ago, 29.6% versus 17.8% for CBD, *P* < .001 (Table 2).

**TABLE 2:** CBD and marijuana use and reporting by zip code rurality.

	CBD			Marijuana		
	Rural	Urban	Total	Rural	Urban	Total
	No. (%)	No. (%)	No. (%)	No. (%)	No. (%)	No. (%)
Ever tried or taken a product	233 (45.1)	247 (46.8)	480 (48.8)	296 (57.5)	325 (61.9)	**621** **^++^** **(59.7)**
Within the past year	143 (27.7)	162 (30.7)	305 (31.0)	132 (25.6)	**181** ^**^ **(34.5)**	313 (30.1)
Longer than a year ago	90 (17.4)	85 (16.1)	175 (17.8)	164 (31.8)	144 (27.4)	**308** **^++^** **(29.6)**
Told health care professional that you have tried or taken (of those who had tried or taken)	99 (47.1)	**128** ^*^ **(56.6)**	227 (52.1)	133 (47.7)	**183** ** **(59.6)**	316 (53.9)

* Significant by zip code population density at *P*<.05.

** Significant by zip code population density at *P*<.01.

^++^ Significant by CBD/marijuana at *P*<.01.

Logistic regression produced significant odds ratios by gender, rurality, age, and race/ethnicity. Being older (65+) was negatively associated with having heard of CBD (OR = 0.489; 95% CI: 0.277‐0.864, *P* = .014). Having a race/ethnicity that was not White, non‐Hispanic was also negatively associated with CBD familiarity (OR = 0.518; 95% CI: 0.278‐0.968; *P* = .039). Having a race/ethnicity that was not White, non‐Hispanic was positively associated with having told a health care professional about CBD use for those with prior use (OR = 1.724; 95% CI: 1.109‐2.916; *P* = .042). Finally, living in an urban zip code was positively associated with having told a health care professional about marijuana use for those with prior use (OR = 1.493; 95% CI: 1.057‐2.110; *P* = .023), as was having a race/ethnicity that was not White, non‐Hispanic (OR = 1.607; 95% CI: 1.004‐2.571; *P* = .048). Increasing age was negatively associated with marijuana use disclosure (OR = 0.975; 95% CI: 0.965‐0.986; *P* < .001) (Table 3). While there was a significant difference in reporting marijuana use between rural and urban respondents, there was no significant difference in knowledge or reporting of CBD.

**TABLE 3:** Logistic regression odds ratios for significant outcomes.

Covariates	Heard of CBD	Told health care professional about CBD use	Told health care professional about marijuana use
Gender: Women (vs Men)	1.428 [0.841, 2.425]	0.688 [0.468, 1.013]	0.809 [0.577, 1.134]
Zip Code Population Density: Urban (vs Rural)	0.621 [0.355, 1.085]	1.306 [0.886, 1.926]	**1.493** * **[1.057, 2.110]**
Age: Year	–	1.001 [0.989, 1.013]	**0.975** ^**^ **[0.965, 0.986]**
Age: 65+ (vs 18‐64)	**0.489** ^*^ **[0.277, 0.864]**	–	–
Race: Not White, non‐Hispanic (vs White, non‐Hispanic)	**0.518** ^*^ **[0.278, 0.968]**	**1.724** ^*^ **[1.109, 2.916]**	**1.607** ^*^ **[1.004, 2.571]**
Constant	25.333	0.988	3.142
N	1,044	436	586

*Note*: 95% confidence intervals for reported odds ratios are in brackets.

* *P* < .05.

** *P* < .01.

## DISCUSSION AND CONCLUSIONS

State‐legal cannabis has expanded substantially in the United States since 1996. However, the complicated nature of federal prohibition mixed with variation in state laws and remaining social stigma has yielded myriad problems for users, industry, and governments. We focus here specifically on the vital doctor‐patient relationship and patient reticence in informing their health care providers about their cannabis use, regardless of whether that use is legal or not. We clearly find that, while there is no difference between rural and urban Pennsylvanians in their willingness to discuss CBD use, rural residents are less likely than urban residents to report marijuana use. This is notable in a state where medical cannabis is legal, but recreational adult use is not.

While questions related to stigma were not directly asked, these results comport with the notion that lingering stigma still affects medical cannabis users. Perceived and expressed stigma, including within the health care system, remains considerable even after nearly 30 years of steady legalization in the United States.^33^ Notably, even in Germany, where medical cannabis has been reimbursable by insurance companies since 2017, medical students do not exhibit competence in prescribing medical cannabis therapy.^34^ In the United States, there is recognition among medical school faculty and deans of the need to teach medical students about cannabis therapy—as opposed to simply treating cannabis as an illicit substance with no medical efficacy—but deployment of such education remains sparse.^35^ This means that the onus remains on physicians to educate themselves on both the emerging research on medical efficacy and the potential harms of medical cannabis.

Both a lack of physician awareness and patients withholding cannabis use from one's primary care doctor and other medical professionals can have serious consequences. A lack of physician awareness means that patients are also on their own in seeking answers to using medical cannabis for treatment.^36^ While they may receive some standardized training in certain states, budtenders at dispensaries are not physicians and tend to focus more on the type of high a patient desires rather than proper dose and form.^37^ Patients withholding information about cannabis use can be problematic for implementing the holistic care that is expected from a PCP, who is expected to coordinate care across different specialties and treatments to which a patient may be prescribed. If a physician is not aware of cannabis use, they may miss the potential for negative side‐effects, drug interactions, and failed attempts at weaning off traditional medicines.^8^, ^38^

The problems of stigma, and the consequences of withholding use from one's physician, appear to be compounded for rural Americans. In a context where both physician and dispensary access are more limited, and where there are closer relationships between patients and providers outside the doctor's office, rural residents appear less likely to report cannabis use to their physicians. This comports with other groups (eg, older adults, veterans, and more) that report both greater perceived stigma and withholding use from not only doctors, but even friends and family.^39^, ^40^ Additionally, during the survey period, urban populations were in closer proximity to dispensaries in New York and New Jersey, which had launched adult‐use recreational programs. This would be compounded after the survey period by the launch of adult use in Maryland (July 2023) and Ohio (August 2024). Closer proximation to cross‐border adult‐use dispensaries likely further advantages access for urban Pennsylvanians. This can also help to explain why more urban respondents reported past year use than rural, given the timeline of sales starting in New York and New Jersey relative to when the survey was fielded.

This study is a starting point for focusing attention on cannabis stigma and the doctor‐patient relationship among rural Americans. It certainly has limitations in that it focuses on a single state and does not capture perceived stigma or more depth about the doctor‐patient relationship. Such depth was not possible with an omnibus statewide survey; however, the insights are no less valuable. Additional research focused on rural populations should conduct more focused surveying as well as deeper interviews with rural patients and doctors to better understand their needs and concerns. Another limitation is that we do not differentiate medical or recreational cannabis use or the frequency of use when asking respondents if they have reported use to their physicians. It is possible that reporting behavior is higher for more‐frequent use or for medical use, which is legal in Pennsylvania, than recreational, which remains prohibited. Additional research can work to untangle the 2 uses. Finally, the low response rate is a limitation with the possibility that those motivated to participate do not accurately reflect the broader population of Pennsylvania.

## CONFLICT OF INTEREST STATEMENT

The authors declare no conflicts of interest.

## FUNDING INFORMATION

The survey was funded by in‐kind support from the Institute for State and Regional Affairs at Penn State Harrisburg.

## ENDNOTES

i PA State Data Center. 2023. Urban Area Changes: An Overview of Changes in Urban Areas from 2010 to 2020. Institute for State & Regional Affairs at Penn State Harrisburg.

ii UDS Mapper. 2023. ZIP Code to ZCTA Crosswalk. American Academy of Family Physicians. Health Resources & Services Administration (HRSA), Uniform Data System (UDS). https://udsmapper.org/
